# Peri-Pandemic Acceptance of Influenza and COVID-19 Vaccination by Swiss Healthcare Workers in Primary Care 2020/21: A Cross-Sectional Study

**DOI:** 10.3389/ijph.2023.1605832

**Published:** 2023-11-15

**Authors:** Olga Morgel, Astrid Czock, Phung Lang

**Affiliations:** ^1^ Interdisciplinary Emergency Centre Lucerne Cantonal Hospital, Lucerne, Switzerland; ^2^ QualiCCare, Baden, Switzerland; ^3^ Epidemiology, Biostatistics and Prevention Institute at the University of Zurich, Zurich, Switzerland

**Keywords:** healthcare workers, primary care, COVID-19 vaccination, behaviour, influenza vaccination

## Abstract

**Objectives:** To assess and compare influenza and COVID-19 vaccination uptake of Swiss healthcare workers (HCWs) in primary care 2020/21.

**Methods:** Influenza and COVID-19 vaccination uptake and recommendation behaviours of HCWs in the primary care were assessed using an online semi-structured questionnaire. Associations between vaccination rates and age, language, gender, profession, vaccination history, vaccination training and recommendation behaviours were evaluated using descriptive and multivariable logistic regression analyses.

**Results:** Vaccinated against COVID-19 in 2020/21 were 91.8% of the 1,237 participating HCWs, while 60.1% were vaccinated against influenza. Physicians and pharmacists presented the highest influenza vaccination rates (87.3%, 73.7%, respectively) compared to nurses (45.8%) and medical practice assistants (52.5%) while COVID-19 vaccination rates were high across all professions. Influenza and COVID-19 vaccination rates were significantly associated with age, profession, vaccination history, vaccination training and recommendation behaviours.

**Conclusion:** Acceptance for influenza vaccination has increased during the pandemic but is lower than that for COVID-19 among the HCWs. Demographics, vaccination status and vaccination training impact the vaccination behaviour among HCWs and should be considered in future campaigns to increase vaccination uptake.

## Introduction

Influenza remains one of the world’s greatest public health challenges, leading to an estimated 1 billion cases worldwide, of which 3 to 5 million are severe and resulting in 290,000 to 650,000 influenza-related respiratory deaths [1]. The World Health Organization recommends seasonal influenza vaccination to high-risk groups (the elderly, people of any age with chronic medical conditions), persons having family or regular personal contact to high-risk group, as well as pregnant women and children under 5 years [2]. In addition, it is recommended for all healthcare workers (HCW), as well as staff in nurseries, day-care centres and nursing homes, including healthcare students and interns [3]. In 2009, the Council of the European Union (EU) formulated its recommendations on influenza vaccination aiming to achieve a 75% coverage among at-risk groups by 2015 [4]. Despite this recommendation and the potential dangers of the disease, influenza vaccination rate (IVR) among healthcare workers in Switzerland and elsewhere, is suboptimal [5–7]. Surveys by the Swiss Federal Office of Public Health (FOPH) revealed a 23% IVR in HCWs during the season 2018/19 [6] and about 26% in 2020/21 [5].

In 2020, the COVID-19 pandemic, caused by the severe acute respiratory syndrome coronavirus-2 (SARS-CoV-2), became a global challenge, especially in the healthcare sector [8]. Worldwide, according to Johns Hopkins University, from 05 January 2020 until 30 January 2023 over 752 million people have been infected with SARS-CoV-2, and over 6.83 million have died from it in 2020/21 [9]. In the EU and European Free Trade Association countries, there were more deaths in 2020 (551,000 death) than the annual average for 2016–2019, as the excess mortality rate reached its highest level in early April 2020 (141% compared to baseline) [10]. According to the FOPH, there were more than 4,170,683 laboratory-confirmed cases of COVID-19 disease in Switzerland and Liechtenstein from 8 June 2020 to 17 October 2022, of which 11,972 died from or with COVID-19 [11]. With 76,195 deaths, the mortality rate in 2020 exceeded the average annual deaths by 12.2% [12].

In addition to various mitigation efforts, such as lockdowns, face-masks, social distancing and hand hygiene, the new COVID-19 vaccines, licenced in December 2020 in Switzerland [13], were promoted as an effort to contain the virus. HCWs with patient contact and caregivers of elderly and chronically ill patients were in the second priority group for access to the vaccination [14].

Studies worldwide show that acceptance of influenza and COVID-19 vaccination depends predominantly on demographic, socioeconomic, and health status factors [15–17]. Other factors resulting in vaccine hesitancy include misinformation, misconception of perceived risk, fear of side effects, mistrust of the pharmaceutical companies and influence of social networks [7, 18, 19]. Studies in Switzerland on the COVID-19 vaccination acceptance showed a cautious, partly sceptical attitude of HCWs towards the licensed vaccines [20, 21]. They revealed that approx. 40% HCW were willing to be vaccinated against COVID-19 shortly before the vaccine was available.

Our study aimed to evaluate IVR, recommendations and reasoning by Swiss HCWs in primary care during the COVID-19 pandemic in 2020/21. Furthermore, we assessed COVID-19 vaccination rate (CVR), recommendations and reasoning and compared these results with the respective influenza results. To assess the possible impact of the pandemic on IVR, we replicated the methodology, sampling design and sampling of the pre-pandemic cross-sectional survey from 2015/16 [22].

## Methods

We hypothesize that the IVR among HCWs in primary care participating in 2015/16 is different from those in 2020/21. Additionally, we hypothesize that there is a difference between CVR and IVR among HCWs participating in 2020/21.

### Data Collection

This cross-sectional study was conducted between November 2021 and February 2022 using a online-questionnaire available in three of the four national Swiss languages: German, French and Italian. To ensure comparability with the 2016 survey [22], the same method of data collection was used and the same target population was addressed.

The target population included HCWs in primary care, namely physicians, pharmacists, nurses, medical practise assistants (MPA) and pharmacy technicians as well as public health officers. Potential participants were recruited by participating national professional societies, healthcare leagues and medical networks (Health Maintenance Organisation), who sent a survey link via direct mailing or newsletter to their members. Two reminders were sent. Participation was voluntary and data obtained was fully anonymized. An inquiry to the Ethic Commission of the canton Zürich confirmed that no ethical approval was required (Basec No. 2021-01316).

### Questionnaire

To allow a direct comparison to the results of the 2015/16 survey, the same questions regarding influenza vaccination were used [22]. For COVID-19 vaccination, the question template was replicated. The semi-structured questionnaire was developed originally in German by the QualiCCare working group “Influenza vaccination for NCD-patients” and was pre-tested in German for clarity, comprehension and accuracy, after which it was professionally translated into French and Italian, and reviewed by native speaking experts in the working group and others.

The questionnaire, included in the Supplementary Material, was divided into a demographics section, followed by a section concerning influenza and COVID-19 vaccinations. Furthermore, participants were asked whether they were trained to vaccinate. Multiple answers were allowed in the questions concerning reasons for accepting or refusing vaccination and their recommendation; the participants could also add comments in the free-text box for these questions. IP-traceability was suppressed in the Survey Monkey tool, so that, completed questionnaires could neither be retracted nor erased.

### Analysis

The raw data from the survey was quantified into the following categories: profession, sex, language, age, status of vaccination training as well as vaccination status, reasons for and against being vaccinated, how often vaccination recommendations were given and underlying reasons for both the influenza and COVID-19 vaccinations. Only cases with complete data were retained for analysis. Language was defined according to the language of the completed questionnaire. Profession was collapsed from 11 to 6 categories and workplace from 11 to 5 categories. For the question pertaining to vaccination training, the four possible responses were collapsed to binary responses by merging yes with “I am currently in training” and no with “I am deciding”. Pearson Chi-squared tests of independence or Fisher’s exact test were performed to evaluate the associations between influenza vaccination status and HCW profession, age, language, vaccination history, status of vaccination training and the prospect of recommending influenza vaccination; similar tests were conducted regarding COVID-19. 2 × 2 continency tables were created to compare data from the 2015/16 survey [22] with the current survey. A multivariable logistic regression analysis was performed to evaluate the odds of being vaccinated against influenza and COVID-19, respectively, and sex, age, language and profession; crude and adjusted odds ratios (OR) and 95% confidence intervals (CI) were calculated. A *p*-value < 0.05 was considered significant. Data analysis was performed using STATA (Version 17.0).

## Results

### Participant Characteristics

Of 1,378 HCWs who completed the questionnaire, 1,237 cases were kept for analysis after data cleaning. Excluded from the analysis were 93 HCWs who reported the hospital as their place of work and 48 HCWs did not answer all questions relevant to the analysis.

Participation among pharmacists (33.8%) was highest, followed by MPA (27.9%) then by physicians (16.9%) and finally nurses and pharmacy technicians (4.8% and 4.9%) (Table 1).

**TABLE 1:** Vaccination rates and recommendation for influenza and COVID-19 vaccinations among healthcare workers, detailed by profession, vaccination training and influenza season (Switzerland 2020/21).

		Profession, *n* (%)						
Demographics	Physician	Pharmacist	Nurses	MPA	Pharmacy technician	Others^a^	*p*-value	Total, *n*
Influenza season								
2015/16 [22]	151 (14.2)	433 (40.9)	110 (10.4)	100 (9.4)	140 (13.2)	123 (11.6)	—	1,057
2020/21	209 (16.9)	418 (33.8)	59 (4.8)	345 (27.9)	60 (4.9)	146 (11.8)	—	1,237
Workplace								
Private practise	200 (95.7)	—	8 (13.6)	325 (94.2)	—	10 (6.8)	—	543 (43.9)
Pharmacy	1 (0.5)	394 (94.3)	1 (1.7)	—	53 (88.3)	6 (4.1)	—	455 (36.8)
Health league	1 (0.5)	1 (0.2)	8 (13.6)	—	—	61 (41.8)	—	71 (5.7)
Homecare	—	—	28 (47.5)	—	—	6 (4.1)	—	34 (2.8)
Others	7 (3.3)	23 (5.5)	14 (23.7)	20 (5.8)	7 (11.7)	63 (43.1)	—	134 (10.8)
Vaccination training	181 (86.6)	300 (71.8)	27 (45.8)	303 (87.8)	18 (30.0)	20 (13.7)	<0.001	849 (68.6)
Yes								
Vaccination uptake								
Influenza 2015/16 [22]	118 (78.1)	205 (47.3)	32 (29.0)	13 (13.0)	34 (24.2)	23 (19.4)	<0.001	425 (40.2)
Vaccinated								
Recommend^b^	101 (67.3)	264 (61.8)	62 (57.4)	42 (43.3)	38 (27.5)	40 (33.3)	<0.001	547 (52.6)
Influenza 2020/21								
Vaccinated	179 (85.6)	305 (73.0)	27 (45.8)	181 (52.5)	22 (36.7)	29 (19.9)	<0.001	743 (60.1)
Recommend^b^	135 (64.6)	324 (77.5)	36 (61.0)	241 (69.9)	27 (45.0)	37 (25.3)	<0.001	800 (64.7)
Influenza 2021/22^c^								
I plan to get vaccinated	176 (84.2)	285 (68.2)	27 (45.8)	146 (42.3)	21 (35.0)	31 (21.2)	<0.001	686 (55.5)
COVID-19 2020/21								
Vaccinated	200 (95.7)	396 (94.7)	51 (86.4)	309 (89.6)	52 (86.7)	128 (87.7)	0.002	1,136 (91.8)
Recommend^b^	160 (76.6)	372 (89.0)	43 (72.9)	266 (77.1)	38 (63.3)	57 (39.0)	<0.001	936 (75.7)

*n* = number of participants.

^a^ Others Profession: psychosocial therapist, administration, public health specialist, social worker, other healthcare profession.

^b^ For the variable recommend, there were four possible answers (yes, no, no patients, only in certain cases), but only the responses for “yes” are displayed. *p*-values are calculated for all four possible answers.

^c^ For the variable Influenza 2021/22, there were three possible answers (yes, no, do not know), but only the responses for “yes” are displayed. *p*-values are calculated for all three possible answers.

The questionnaires were answered by 67.4% of the HCW in German, 29.8% in French and 2.8% in Italian (Table 2). Differences can be seen in sex and age with participation in the survey being higher among women (84.3%) (Table 2) and those aged 31–60 years (total 79.3%, Table 3). The majority of the participants were between 31–60 years of age, with a median age of 47 [Interquartile Range (IQR) 18–65] (Table 3), and by profession pharmacists (33.8%) and MPAs (27.9%) (Table 1).

**TABLE 2:** Vaccination rates and recommendation for influenza and COVID-19 vaccinations among healthcare workers, detailed by language region, sex, vaccination training and influenza season (Switzerland 2020/21).

Demographics									
Influenza season	Sex^a^, *n* (%)				Language, *n* (%)				
	f	m	d	*p*-value	DE	FR	IT	*p*-value	Total, *n*
2015/16 [22]	793 (75.0)	264 (25.0)	—	—	713 (67.5)	289 (27.3)	55 (5.2)	—	1,057
2020/21	1,043 (84.3)	190 (15.4)	4 (0.32)	—	834 (67.4)	368 (29.8)	35 (2.8)	—	1,237
Swiss Population 2021, % [23, 24]	50.3	49.7	—		62.3	22.6	8.3		8,738,791
Vaccination training									
Yes	720 (69.0)	129 (67.9)	—	0.756	553 (66.3)	270 (73.4)	26 (74.3)	0.040	849 (68.6)
Vaccination uptake									Total, *n* (%)
Influenza 2015/16 [22]									
Vaccinated	275 (34.7)	150 (56.9)	—	<0.001	266 (37.3)	139 (48.1)	20 (36.4)	0.006	425 (40.2)
Recommend^b^	380 (48.7)	167 (64.2)	—	<0.001	334 (47.9)	182 (63.2)	31 (57.4)	<0.001	547 (52.6)
Influenza 2020/21									
Vaccinated	604 (57.9)	137 (72.1)	2 (50.0)	<0.001	499 (59.8)	222 (60.3)	22 (62.9)	0.931	743 (60.1)
Recommend^b^	672 (64.4)	127 (66.8)	1 (25.0)	0.414	536 (64.3)	239 (64.9)	25 (71.4)	0.047	800 (64.7)
Influenza 2021/22^c^									
I plan to get vaccinated	548 (52.5)	136 (71.6)	2 (50.0)	<0.001	485 (58.2)	180 (48.9)	21 (60.0)	0.004	686 (55.5)
COVID-19 2020/21									
Vaccinated	955 (91.6)	177 (93.2)	4 (100)	0.637	773 (92.7)	329 (89.4)	34 (97.1)	0.081	1,136 (91.8)
Recommend^b^	790 (75.7)	144 (75.8)	2 (50.0)	0.425	637 (76.4)	269 (73.1)	30 (86.7)	0.003	936 (75.7)

Male (m), female (f), other (d).

^a^ Sex.

^b^ For the variable recommend, there were four possible answers (yes, no, no patients, only in certain cases), but only the responses for “yes” are displayed. *p*-values are calculated for all four possible answers, using Fisher’s exact test.

^c^ For the variable Influenza 2021/22, there were three possible answers (yes, no, do not know), but only the responses for “yes” are displayed. *p*-values are calculated for all three possible answers.

**TABLE 3:** Vaccination rates and recommendation for influenza and COVID-19 vaccinations among healthcare workers, detailed by age group and influenza season (Switzerland 2020/21).

	Age of the participants						Total	
Demographics	16–20	21–30	31–40	41–50	51–60	61+	*n*	*p*-value
Influenza season								
Study 2015/16, *n* (%) [22]	60 (5.7)	203 (19.2)	215 (20.3)	226 (21.4)	274 (25.9)	79 (7.5)	1,057	—
Study 2020/21, *n* (%)	17 (1.4)	128 (10.4)	241 (19.5)	354 (28.6)	386 (31.2)	111 (9.0)	1,237	—
Swiss Population 2021 [23], %	4.8	12.1	14.3	13.7	14.7	9.6	8,738,791	
Vaccination training								
Yes	10 (58.8)	97 (75.8)	180 (74.7)	236 (66.8)	256 (66.3)	70 (63.1)	849 (68.6)	0.049
Vaccination Uptake								
Influenza 2015/16 [22]								
Vaccinated, *n* (%)	4 (6.7)	57 (28.1)	89 (41.4)	92 (40.7)	134 (48.9)	49 (62.0)	425 (40.2)	<0.001
^a^Recommend, *n* (%)	25 (43.1)	88 (44.2)	116 (54.7)	115 (51.8)	152 (56.1)	51 (65.4)	547 (52.6)	0.003
Swiss Population [43], %	6.51	7.71	6.37	7.52	12.26	26.7	25.3	
Swiss HCW [3], %							18	
Influenza 2020/21								
Vaccinated, *n* (%)	2 (11.8)	62 (48.4)	138 (57.3)	203 (57.3)	264 (68.4)	74 (66.7)	743 (60.1)	<0.001
^a^Recommend, *n* (%)	8 (47.1)	92 (71.9)	143 (59.3)	220 (62.2)	260 (67.4)	77 (69.4)	800 (64.8)	0.045
Swiss Population [5], %	—	—	—	—	—	38	—	
Swiss HCW [5], %	—	—	—	—	—	—	26	
Influenza 2021/22								
^b^I plan to get vaccinated, *n* (%)	3 (17.7)	37 (28.9)	122 (50.6)	190 (53.7)	259 (67.1)	75 (67.6)	686 (55.5)	<0.001
COVID-19 2020/21								
Vaccinated, *n* (%)	14 (82.4)	109 (85.2)	220 (91.3)	325 (91.8)	365 (94.6)	103 (92.8)	1,136 (91.8)	0.018
^a^Recommend, *n* (%)	9 (52.9)	100 (78.1)	174 (72.2)	251 (70.9)	314 (81.4)	88 (79.3)	936 (75.7)	0.019
Swiss Population [35], %	49.2	71.5	73.2	77.3	80.5	86.1	70.0	
Swiss HCW [30], %							85.0/+5.0	

^a^ For the variable recommend, there were four possible answers (yes, no, no patients, only in certain cases), but only the responses for “yes” are displayed. *p*-values are calculated for all four possible answers.

^b^ For the variable Influenza 2021/22, there were three possible answers (yes, no, do not know), but only the responses for “yes” are displayed. *p*-values are calculated for all three possible answers.

### Influenza Vaccination Status by Profession, Language, Age and Sex

Overall, the IVR among participating HCW was 60.1% in 2020/21 compared to 40.2% in 2015/16 (*p* < 0.001, Table 1). Physicians presented the highest IVR (85.6%), followed by pharmacists (73.0%) and MPAs (52.5%) (*p* < 0.001, Table 1). 55.5% of all HCWs indicated that they planned to also get vaccinated against influenza in the upcoming 2021/22 season (*p* < 0.001, Table 1).

In 2020/21, there were significant differences between influenza vaccination status, profession, sex and age, but not for language (Tables 1–3). IVR among male participants were 72.1% compared to 57.9% females (*p* < 0.001, Table 2). Similarly, there was a significant difference in IVR across all age groups (*p* < 0.001, Table 3). The intention to get vaccinated in the next influenza season decreased for those ≤50 (47.6%), while it remained equally high for those >51 (67.2%). There was no significant difference in IVR across the different languages in 2020/21 (*p* = 0.931, Table 2). However, fewer French-speaking participants (48.9%) planned to get vaccinated against influenza in 2021/22 while the intention remained the same for the German- and Italian-speaking ones (58.2% and 60%, respectively, *p* = 0.004, Table 2).

We also examined the relationship between status of vaccination training, language, sex, age group, profession and influenza vaccination status in a multivariable logistic regression analysis (Table 4, Part A). Physicians and pharmacists have significantly higher odds of being vaccinated against influenza than nurses [Adjusted Odds Ratio (AOR): 6.10 (95% CI 3.12–11.92, *p* < 0.001) and 3.40 (95% CI 1.90–0.06, *p* < 0.001), respectively]. Compared to participants aged 16–20, all other age groups had significantly higher odds of being vaccinated against influenza, with the highest odds by aged 51–60 (AOR 9.71; 95% CI 2.08–45.24, *p* = 0.004).

**TABLE 4:** Logistic regression analysis during the influenza and COVID-19 season (Switzerland 2020/21).

Part A: Adjusted participants’ characteristics associated with influenza vaccination status									
Characteristics	*n*	Crude odds ratio	95% CI lower	95% CI upper	*p*-value	Adjusted odds ratio	95% CI lower	95% CI upper	*p*-value
Vaccination Training									
No	388	Ref	—	—	—	Ref			
Yes	849	2.7481	2.1466	3.5182	<0.001	1.7710	1.2930	2.4257	<0.001
Pearson chi2(1) = 66.2462 Pr < 0.001									
Language									
German	834	Ref	—	—	—	Ref	—	—	—
French	368	1.0208	0.7946	1.3114	0.872	0.9287	0.6972	1.2369	0.613
Italian	35	1.1361	0.5645	2.2867	0.721	0.5810	0.2703	1.2489	0.164
Pearson chi2(2) = 0.1431 Pr = 0.931									
Sex									
Male	190	Ref	—	—	—	Ref	—	—	—
Female	1,043	0.5323	0.3788	0.7478	<0.001	0.7919	0.5213	1.2025	0.273
Diverse	4	0.3868	0.0531	2.8172	0.349	2.0518	0.2261	18.6191	0.523
Pearson chi2(2) = 13.6713 Pr = 0.001									
Age group									
16–20	17	Ref	—	—	—	Ref	—	—	—
21–30	128	7.0452	1.5477	32.0699	0.012	4.1208	0.8642	19.6486	0.076
31–40	241	10.0482	2.2481	44.9117	0.003	5.1068	1.0881	23.9676	0.039
41–50	354	10.0824	2.2715	44.7525	0.002	5.7683	1.2367	26.9041	0.026
51–60	386	16.2290	3.6542	72.0753	<0.001	9.7069	2.0828	45.2404	0.004
60+	111	14.9996	3.2569	69.0808	0.001	7.1803	1.4678	35.1259	0.015
Pearson chi2(5) = 38.8097 Pr < 0.001									
Profession									
Nurse	17	Ref	—	—	—	Ref	—	—	—
Physician	128	7.0716	3.7223	13.4345	<0.001	6.0991	3.1212	11.9181	<0.001
Pharmacist	241	3.1990	1.8350	5.5768	<0.001	3.3961	1.9015	0.0652	<0.001
MPA	354	1.3080	0.7516	2.2764	0.342	1.3704	0.7554	2.4861	0.300
Pharmacy technician	386	0.6862	0.3295	1.4288	0.314	1.0589	0.4916	2.2806	0.884
Other	111	0.2938	0.1528	0.5649	<0.001	0.3851	0.1957	0.7578	0.006
Pearson chi2(5) = 211.4303 Pr < 0.001									

Part B: Adjusted participants’ characteristics associated with COVID-19 vaccination status									
Characteristics	*n*	Crude Odds Ratio	95% CI Lower	95% CI Upper	*p*-value	Adjusted Odds Ratio	95% CI Lower	95% CI Upper	*p*-value
Vaccination Training									
No	388	Ref				Ref			
Yes	849	1.654	1.0729	2.4623	0.022	1.4787	0.8753	2.4979	0.144
Pearson chi2(1) = 5.3338 Pr = 0.021									
Language									
German	834	Ref	—	—	—	Ref	—	—	—
French	368	0.6657	0.4365	1.0152	0.059	0.6099	0.3864	0.9626	0.034
Italian	35	2.6831	0.3611	19.9363	0.335	1.8555	0.2432	14.1571	0.551
Pearson chi2(2) = 5.0250 Pr = 0.081									
Sex									
Male	190	Ref	—	—	—	Ref	—	—	—
Female	1,043	0.7971	0.4357	1.4582	0.462	1.2622	0.6481	2.4583	0.493
Diverse	4	1	—	—	—	1	—	—	—
Pearson chi2(2) = 0.9022 Pr = 0.637									
Age group									
16–20	17	Ref	—	—	—	Ref	—	—	—
21–30	128	1.2293	0.3222	4.6891	0.762	1.0415	0.2617	4.1447	0.954
31–40	241	2.2448	0.5668	8.4446	0.232	1.8525	0.4727	7.2597	0.376
41–50	354	2.4015	0.6522	8.8427	0.188	2.1599	0.5628	8.2882	0.262
51–60	386	3.7245	0.9927	13.9737	0.051	3.3862	0.8650	13.2554	0.080
60+	111	2.7589	0.6539	11.6396	0.167	2.2016	0.4949	9.7941	0.300
Pearson chi2(5) = 13.7073 Pr = 0.018									
Profession									
Nurse	17	Ref	—	—	—	Ref	—	—	—
Physician	128	3.4858	1.2813	9.4828	0.014	3.4043	1.1909	9.7319	0.022
Pharmacist	241	2.8235	1.1947	6.6733	0.018	3.2692	1.3314	8.0271	0.010
MPA	354	1.3464	0.5922	3.0612	0.478	1.6680	0.6789	4.0977	0.265
Pharmacy technician	386	1.0196	0.3556	2.9235	0.971	1.5810	0.5199	4.8078	0.420
Other	111	1.1154	0.4563	2.7267	0.811	1.4216	0.5615	3.5991	0.458
Pearson chi2(5) = 19.0178 Pr = 0.002									

*n*, number of participants; Total *n* = 1,237; Ref, reference; CI, confidence interval.

### COVID-19 Vaccination Status by Profession, Language, Age and Sex

The overall CVR among the participating HCW was 91.8% during the season 2020/21 with two peaks between May to July and November to December (Table 1), with physicians (95.7%) and pharmacists (94.7%) being best vaccinated (*p* = 0.002). There was no significant difference in CVR in regard to sex (*p* = 0.637) and language (*p* = 0.081) among the participants (Table 2). However, there was a significant association with age (*p* = 0.018; Table 3).

The multivariable logistic regression analysis of COVID-19 vaccination status and status of vaccination training, language, sex, age and profession yielded the same trend as seen with influenza (Table 4, Part B). Physicians and pharmacists have significantly higher odds of being vaccinated against COVID-19 than nurses [AOR: 3.40 (95% CI 1.19–9.73, *p* = 0.022) and 3.27 (95% CI 1.33–8.03, *p* = 0.010), respectively]. However, French-speaking HCWs have significantly lower odds of being vaccinated against COVID-19 than their German-speaking counterparts [AOR: 0.60 (95% CI 0.39–0.96, *p* = 0.034)].

### Reasons for Getting and Not Getting Vaccinated Against the Influenza and COVID-19

Patient protection (81.9%) and personal protection (81.9%) were the main reasons for HCWs to get vaccinated against influenza; in contrast, the main reasons for a vaccination against COVID-19 were personal protection (83.0%), way out of the pandemic (78.8%), family protection (73.2%) and patient protection (71.0%), followed by herd immunity (66.1%), role model (55.4%), social pressure (8.3%) and obtaining a COVID-19 vaccination certificate (27.5%) were further reasons for getting vaccinated against COVID-19 (Supplementary Figure S1). There were no significant differences across the language.

Reasons for not getting vaccinated against the influenza and COVID-19 were more diverse (Supplementary Table S1).

We also asked the HCWs whether a COVID-19 infection influenced their decision to get an influenza vaccination: 70.7% responded no, 27.4% yes and 1.9% did not know, with no difference between sex and language of the HCW (Supplementary Table S2).

### Recommendation of Influenza or COVID-19 Vaccination to Their Patients

Of all the HCWs surveyed, pharmacists (77.5%) were the most likely to recommend the influenza vaccination to their patients, followed by MPA (69.9%), physicians (64.6%) and nurses (61.0%); this distribution is similar for COVID-19 vaccination (89.0%, 77.1%, 76.6% and 72.9%, respectively) (Table 1). The most common reasons for recommending the influenza and COVID-19 vaccinations were patient protection (81.5% and 93.9%, respectively) and conviction (47.0% and 64.3%, respectively). 75.9% HCWs also cited family protection and social pressure as a reason for recommending the COVID-19 vaccination (Supplementary Figure S2; Supplementary Table S3). 5% respondents added new reasons for recommending vaccination such as: herd immunity, way out of the pandemic and solidarity.

There was no difference between sex and recommendation of influenza (*p* = 0.414, Table 2) and COVID-19 (*p* = 0.425, Table 2) vaccinations. In contrast, there was a significant association between language and recommendation of both vaccinations: 86.7% Italian-speaking HCWs recommended the COVID-19 vaccination whereas only 76.4% German- and 73.1% French-speaking HCWs have done that (*p* = 0.003, Table 2); for influenza it was 71.4%, 64.3%, and 64.9%, respectively, *p* = 0.047, Table 2). HCWs aged 21–30 and those >50 also have a significantly higher prospect of recommending influenza (*p* = 0.045, Table 3) and COVID-19 (*p* = 0.019, Table 3) vaccinations than other age groups.

### The Impact of Vaccination Training on HCWs

Table 1 shows that 68.6% of the HCWs surveyed had vaccination training, with the most being physicians (86.6%) and MPA (87.8%), followed by pharmacists (71.8%) while only 45.8% nurses and 30.0% of pharmacy technicians were trained (*p* < 0.001). There was no difference between male (67.9%) and female (69.0%) (*p* = 0.756), but there was a significant difference among the different languages of the participants where 73.4% French-speaking and 74.3% Italian-speaking HCWs had vaccination training compared to 66.3% German-speaking HCW (*p* = 0.040, Table 2). HCWs aged 21–40 received significantly more training than the other age groups (*p* = 0.049, Table 3). The results were similar for COVID-19. In Table 6, we see a significant association between training status of the HCWs and their influenza and COVID-19 vaccination status. 77.4% HCWs who can vaccinate are themselves vaccinated against influenza (*p* < 0.001) and 69.5% against COVID-19 (*p* = 0.021); it was only 22.6% and 30.5%, respectively, for HCWs who had no vaccination training.

There was also an association between vaccination training and vaccination recommendation (Table 5): 73.9% of the respondents who had training in vaccination, always recommended influenza vaccinations to their patients or clients compared to only 44.6% who had no vaccination training (*p*-value < 0.001, Table 5). For the COVID-19 vaccination, it was 82.9%, compared to 59.8% (*p*-value < 0.001, Table 5), respectively.

**TABLE 5:** Recommendations of influenza and COVID-19 vaccinations by healthcare workers, detailed by vaccination status and level of vaccination training (Switzerland 2020/21).

	Total, *n* (%)	Recommend the influenza vaccine				
Influenza vaccinated 2020/21		No	Yes	No patients	In certain cases	*p*-value
No	494 (39.9)	32 (6.5)	218 (44.1)	93 (18.8)	151 (30.6)	
Yes	743 (60.1)	6 (0.8)	582 (78.3)	32 (4.3)	123 (16.6)	
Total, *n*	1,237	38 (3.1)	800 (64.7)	125 (10.1)	274 (22.2)	<0.001

		Recommend the COVID-19 vaccine				
COVID-19 vaccinated 2020/21		No	Yes	No patients	In certain cases	*p*-value
No	101 (8.2%	20 (19.8)	14 (13.9)	22 (1.8)	45 (44.6)	
Yes	1,136 (91.8%)	25 (2.2)	922 (81.2)	98 (8.6)	91 (8.0)	
Total, *n*	1,237	45 (3.6)	936 (75.7)	120 (9.7)	136 (11.0)	<0.001

		Recommend the influenza vaccine				
Vaccination training		No	Yes	No patients	In certain cases	*p*-value
No	388 (31.4%)	19 (4.9)	173 (44.6)	94 (24.2)	102 (26.3)	
Yes	849 (68.6%)	19 (2.2)	627 (73.9)	31 (3.7)	172 (20.26)	
Total, *n*	1,237	38 (3.1)	800 (64.7)	125 (10.1)	274 (22.2)	<0.001

		Recommend the COVID-19 vaccine				
Vaccination training		No	Yes	No patients	In certain cases	*p*-value
No	388 (31.4%)	18 (4.6)	232 (59.8)	89 (2.9)	49 (12.6)	
Yes	849 (68.6%)	27 (3.2)	704 (82.9)	31 (3.7)	87 (10.3)	
Total, *n*	1,237	45 (3.6)	936 (75.7)	120 (9.7)	136 (11.0)	<0.001

*n*, number of participants. Total *n* = 1,237; (%), Percent of participants; no patients, no contact with patients.

### The Impact of Vaccination History on HCWs

In Tables 5, 6, we display the association between vaccination history on vaccination uptake, intent to get vaccinated and recommendation of vaccination for both influenza and COVID-19. Table 6 shows that of the 86.7% respondents who were vaccinated against influenza also intended to get vaccinated again in 2021/22 (*p* < 0.001) and 98.4% (*p* < 0.001) were vaccinated against both influenza and COVID-19. Furthermore, 78.1% who were not vaccinated against the influenza in 2020/21 will also remain unvaccinated against influenza in 2021/22. In contrast, 82.0% of the respondent who were not vaccinated against the influenza, were vaccinated against COVID-19. A significant association between the respondents’ vaccination status and the prospect of recommending either influenza or COVID-19 vaccination was also observed (*p*-value < 0.001; Table 5): among the respondents who were vaccinated against influenza, 78.3% recommended the vaccination to their patients or clients whereas only 44.1% of the non-vaccinated respondents did. This difference was even more evident for COVID-19 among those vaccinated, with 81.2% recommending the COVID-19 vaccine, and only 13.9% of non-vaccinated HCWs did.

**TABLE 6:** Vaccination status of healthcare workers related to level of vaccination training and vaccination history (Switzerland 2020/21).

Training status	Influenza vaccination, *n* (%)				COVID-19 vaccination, *n* (%)		
	Yes	No	Total		Yes	No	Total
Yes	575 (77.4)	274 (55.5)	849 (58.6)		790 (69.5)	59 (58.4)	849 (68.6)
No	168 (22.6)	220 (44.5)	388 (31.4)		346 (30.5)	42 (41.6)	388 (31.4)
Total	743 (60.1)	494 (39.9)	1,237 (100)		1,136 (91.8)	101 (8.2)	1,237 (100)
	Pearson chi2(1) = 66.2462 Pr < 0.001				Pearson chi2(1) = 5.3338 Pr = 0.021		

Influenza vaccinated 2020/21	Intention to get the Influenza Vaccination 2021/22, *n* (%)				COVID-19 Vaccination, *n* (%)		
	Yes	No	Do not know	Total	Yes	No	Total
Yes	644 (86.7)	61 (8.2)	38 (5.1)	743 (60.1)	731 (98.4)	12 (1.6)	743 (60.1)
No	42 (8.5)	386 (78.1)	66 (13.4)	494 (39.9)	405 (82.0)	89 (18.0)	494 (39.9)
Total	686 (55.5)	447 (36.1)	104 (8.4)	1,237 (100)	1,136 (91.8)	101 (8.2)	1,237 (100)
	Pearson chi2(1) = 752.4898 Pr < 0.001				Pearson chi2(1) = 106.4468 Pr = <0.001		

*n*, number of participants. % Percent of participants. Total *n* = 1,237.

## Discussion

Our study showed that 60% of our participants from the primary care sector were vaccinated against influenza during the flu season 2020/21. Compared to the study conducted in 2015/16, this was a 20-percentage-point increase, from 40% to 60% (*p* < 0.001); this difference should be taken with caution as the demographic structure in terms of age, sex, profession of the two study populations in the cross-sectional studies were not identical [22]. Our results were higher than those obtained by the FOPH for influenza seasons 2019–2022, which were each well below 30% [5, 25]. These discrepancies are due to different study designs (telephone survey vs. online survey), different target populations (undefined HCW vs. only HCW involved in primary care) and time of data collection (March vs. November–February). Although we did not find any international publications concerning IVR in HCWs in primary care, they seemed to increase during the pandemic period [26–30]. IVR ranged from 53.6% in a multicentre observational study conducted in Italy [26], to 60.2% from an anonymous online cross-sectional survey conducted in Germany [27], to 78% according to an online survey from Ireland [28].

The significant positive trend in vaccination readiness among HCWs detected between the two compared surveys before (2015/16) and during (2020/21) the pandemic also continues into 2021/22 as the intention to be vaccinated remains high at 56%. Our study also confirmed that a history of influenza or COVID-19 vaccination is a strong predictor of current and future vaccination uptakes and recommendations, as has been shown in 2015/16 and other studies [22, 31, 32]. A recent study by Li et al. (2022) found that the COVID-19 pandemic was positively associated with getting an influenza vaccination [33]. The increase may be due to the desire for personal protection upon encounter with suspected or confirmed COVID-19 patients [34], as symptoms of the two respiratory diseases are similar, as well as to increase patient safety. 27% of respondents from our study admitted that the COVID-19 pandemic influenced their decision to get vaccinated against influenza. Fear of the inability to work was given by several respondents as a reason for precautionary influenza vaccination.

Our results showed that 92% of HCWs were vaccinated against COVID-19, which was higher than those of the general population and confirmed by other studies [30, 35]. The Swiss Medical Society’s survey of physicians’ willingness to vaccinate against COVID-19 revealed that 85% of the primary care physicians in Switzerland had already been vaccinated in June/July 2021, and 5% planned to be vaccinated by the end of the same year [30]. Published data from the German Central Institute for Statutory Health Insurance Physicians [36] on the Corona vaccination campaign showed that CVR was 97.7% among physicians in primary care and 90.4% among non-physician staff, which were significantly higher than the general population average for the corresponding age group [29].

### Vaccination Status by Profession, Language and Age

Our survey confirmed that participating physicians and pharmacists were better vaccinated against influenza and COVID-19 than other HCWs—this corresponds to the results from 2015/16, along with more current research [22, 33, 37–39], while nurses’ and MPAs’ vaccination rates corresponded to the general population [39, 40]. The higher vaccination propensity of physicians and pharmacists may be associated with both a higher level of education and a more available vaccination training in their medical education curriculum [41]. Despite nurses and MPA also being trained to vaccinate, studies have shown their hesitation regarding vaccinations [42, 43]. However, IVR for nurses and MPA have increased significantly from 29% to 13% in 2015/16 to 48% and 51% in 2020/21, respectively. The Swiss Health Survey 2017 also revealed a positive association of vaccination uptake with a healthcare profession and/or an underlying chronic disease, in addition to other known factors [44]. The OKaPII study of the Robert Koch Institute showed an increase in IVR among German physicians from 59% in 2017/18 to 79% in the 2019/20 influenza season and from 31% to 47% for nurses, respectively [45].

In contrast to the 2015/16 survey [22] and other vaccination studies in Switzerland [16, 44], IVR in 2020/21 had no association with the language of the HCWs. This phenomenon could be an influence of the pandemic. IVR and CVR were equally high for all three languages in 2020/21, but the intention to vaccinate in 2021/22 was lowest among the French-speaking HCWs, but which matches pre-pandemic estimates. This contradicts the previously published association between language and vaccination uptake in Switzerland [22, 44, 46] and warrants further investigation.

Taken alone, age continues to be positively associated with influenza and COVID-19 vaccination uptake among HCWs, as also confirmed in other national and international studies [15, 17, 21, 22, 33, 37]. This is also confirmed in the multivariable logistic regression for influenza, but for COVID-19 vaccination, age was no longer a predictor of getting vaccinated during the 2020/21 season. This result again shows an overreaching impact of the pandemic on vaccination uptake [31, 47].

### Reasons for and Against Vaccination

Main personal motives for vaccination among HCW such as self-protection, patient protection, and protection of family members have been confirmed in this study, in the study from 2015/16 and others [17, 22, 33, 38, 48–50]. During the COVID-19 pandemic additional reasons for getting vaccinated were also common, such as herd immunity/solidarity, way out of the pandemic, obtaining a COVID-certificate, and social pressure [27]. In the current survey, solidarity was frequently mentioned as a reason for vaccination against both viral infections, even though, this was expressed differently: some giving priority to elderly and at-risk patients for influenza vaccinations in view of vaccine shortages, but also getting vaccinated as a way for society to exit the pandemic.

The most common reasons for refusal of influenza vaccination included not belonging to the risk group, trust in the immune system, and doubts about the effectiveness of the vaccine [49, 51–54]. In addition to those mentioned above, reasons for refusal and hesitation about COVID-19 vaccination included lack of awareness of the risk of disease and doubts about the safety of the vaccine, because the vaccine was newly developed and launched very quickly, and fears that it was unnecessary [34, 55]. Our results also showed that non-vaccinated HCWs recommended influenza vaccination (44.1%) to their patients significantly more often than COVID-19 vaccination (13.9%), which may reflect more trust in the longstanding influenza than the new COVID-19 vaccine [46, 50, 56].

### Influence of Vaccination Training on HCW Vaccination Status and Recommendations

Our survey shows that training in vaccination was highly correlated with vaccination uptake and recommendation to their patients, which was also observed in another study [50]. Only 46.6% of the responding nurses stated they were able to vaccinate. These results contradict the answers from another Swiss survey among all cantonal health authorities which claimed that nurses are generally authorized to vaccinate in delegation of a physician, while one canton even authorized nurses to vaccinate with full autonomy [57]. This could be due to a misunderstanding of the expression “vaccination training” in our questionnaire or the fact that the nurses neither vaccinate nor trust themselves to vaccinate.

In view of the revealed positive association of vaccination training and vaccination status, optimizing vaccination training among HCWs could result in better understanding, more in-depth information about vaccinations and, consequently, possible higher vaccination acceptance. This could be a reason for the stark increase of the vaccination uptake and acceptance among pharmacists compared with 2015/16, when the first Swiss cantons authorized trained pharmacists to vaccinate against influenza and other infectious diseases [58]. At the time of this survey, 25 of 26 cantons permitted trained pharmacists to vaccinate while trained pharmacy technicians were permitted to vaccinate against COVID-19 in two cantons. The fact that more pharmacists are getting trained in vaccination may plausibly account for the increasing vaccination uptake among pharmacists and pharmacy technicians as well as for the increased prospect of recommending vaccinations.

### Limitations

The major limitation of this study is that it may not be representative of the general HCW population working in the primary care setting in Switzerland and thus generalization of the results is limited. The exact sample size of the participants (n) and thus the response rate cannot be established as we do not know whether the recipients received, opened or read the direct e-mailings or newsletters sent by their participating health organizations. As the interprofessional non-profit association QualiCCare is invested in increasing the quality of care of chronic patients in the primary care setting in Switzerland, its member organizations might be more aware of best practise recommendations and thus, answers of recipients of these direct e-mailings and newsletters could be positively skewed. Additionally, there could be selection bias, as HCWs interested in the topic, were more likely to participate in the survey. As there is a tendency for more females to be employed in the healthcare sector and they participate more often than men in health studies, our study population was also skewed towards females; however, recent publications calculated that, although it did not encompass all HCW included in the sampling frame, there are 87.2% female HCW (not including doctors, pharmacists and pharmacy technicians) working in nursing/assisted living and retirement homes; there are 41.2% female doctors working in a private practice and 77% of all practicing pharmacists were female [59–61]. Furthermore, although this study targeted HCWs in the primary care sector, 93 participants declared that they worked in a hospital. Information on how they obtained the survey link is not available, but they have been removed from the study. Moreover, as this is a cross-sectional study, association and, not causation can be determined. Finally, since influenza vaccination is not routinely recorded in vaccination records, we relied on the participants’ self-report, which could lead to recall and selection biases [62]. For COVID-19 vaccination, we expect very low recall bias as it was relatively new and participants could easily review their vaccination certificate via the readily available and popular COVID-19 app [63].

Another limitation concerns the questionnaire as despite having been pretested, the question regarding the vaccination training could be misunderstood and could produce biased results. It is not clear if respondents understood the vaccination training as the practical training of the vaccination act or the theoretical aspect of vaccinations in general. This could be an explanation that some HCWs claimed to not having been trained in vaccination in spite of this being part of their professional curriculum.

### Conclusion

Swiss HCWs in primary care participating in our study have higher IVR and CVR than the published data for the general population in Switzerland. Moreover, IVR seems to have increased from the pre- to peri-pandemic phases in our study population. HCW tended to be better vaccinated against COVID-19 than influenza, with little variations across professions. The same observations apply for recommendations of vaccination. However, more HCW tended to be vaccinated against COVID-19 than their tendency to recommend these respective vaccinations to their patients, which is the opposite with influenza. The pandemic may have an overreaching impact on vaccination behaviour while also confirming new reasons to get vaccinated, such as solidarity, herd immunity and COVID-certification. Vaccination history and vaccination training play an important role in HCW’s current and future vaccination uptake as well as recommendation of vaccination to their patients. Taken together, these factors can be used to develop new vaccination campaigns and strategies to increase vaccination uptake for the HCWs and ultimately, the general population.

As our results only show vaccination uptake in Swiss primary care settings, this survey should therefore be repeated in hospital settings for a more encompassing overview of HCW vaccination uptake and behaviours in Switzerland.

## References

[1] World Health Organization. Who Launches New Global Influenza Strategy. Geneva, Switzerland: World Health Organization (2019). Available From: https://www.who.int/news/item/11-03-2019-who-launches-new-global-influenza-strategy (Accessed September 15, 2023).

[2] World Health Organization. Seasonal Influenza Vaccines: An Overview for Decision-Makers (2020). Available From: https://apps.who.int/iris/bitstream/handle/10665/336951/9789240010154-eng (Accessed September 15, 2023).

[3] Federal Office of Public Health. Impfen Gegen Grippe (2019). Bern Available From: https://impfengegengrippe.ch/de-ch/impfung/impfempfehlungen.html (Accessed September 15, 2023).

[4] Council of the European Union. Council Recommendation of 22 December 2009 on Seasonal Influenza Vaccination. Official J Eur Union (2009) L348 (1019) 71. Available From: https://eurlex.europa.eu/LexUriServ/LexUriServ.do?uri=OJ:L:2009:348:007 (Accessed September 17, 2023).

[5] Federal Office of Public Health. Vaccination Data From Swiss Federal Health Office. “Bericht zur Grippesaison 2020/21 - bag.Admin.ch.” (2020). Available From: https://www.bag.admin.ch/dam/bag/de/dokumente/mt/infektionskrankheiten/grippe/saisonbericht-grippe-2020/21.pdf.download.pdf/saisonbericht-grippe-2020/21.pdf (Accessed September 17, 2023).

[6] Federal Office of Public Health. Bericht zur Grippesaison 2018/19 (2019). Bern Available From: https://www.bag.admin.ch/dam/bag/de/dokumente/mt/infektionskrankheiten/grippe/saisonbericht-grippe-2018-19.pdf.download.pdf/saisonbericht-grippe-2018-19-de.pdf (Accessed September 17, 2023).

[7] To KW Lai A Lee KCK Koh D Lee SS. Increasing the Coverage of Influenza Vaccination in Healthcare Workers: Review of Challenges and Solutions. J Hosp Infect (2016) 94(2):133–42. 10.1016/j.jhin.2016.07.003 27546456

[8] World Health Organization. WHO Grippe Sensibilisierungskampagne (2023). Available From: https://www.euro.who.int/de/health-topics/communicable-diseases/influenza (Accessed September 17, 2023).

[9] Mathieu E Ritchie H Rodés-Guirao L Appel C Gavrilov D Giattino CH Research and Data: Coronavirus (Covid-19) Cases. Our World in Data (2020). Johns Hopkins University Available From: https://ourworldindata.org/covid-cases (Accessed September 17, 2023).

[10] Eurostat. Weekly Deaths in 31 European Countries (2022). Available From: https://ec.europa.eu/eurostat/statistic-explained/index.php?title=Weekly_death_statistics#Around_1.5_million_additional_deaths_between_January_2020_and_August_2022 (Accessed September 17, 2023).

[11] Federal Office of Public Health. Epidemiologischer Verlauf, Schweiz und Lichtenstein. Information zur Aktuellen Lage, stand 18.10.2022 (2022). Bern Available From: https://www.covid19.admin.ch/de/epidemiologic/death?time=phase2 (Accessed September 17, 2023).

[12] Swiss Federal Statistical Office. Todesursachenstatistik. Covid-19 war 2020 die Dritthäufigste Todesursache in der Schweiz (2020). Bern Available From: https://www.bfs.admin.ch/bfs/de/home/statistiken/kataloge-datenbanken/medienmitteilungen.assetdetail.23284854.html (Accessed September 17, 2023).

[13] Swissmedic. Swissmedic Erteilt Zulassung für den Ersten Covid-19-Impfstoff in der Schweiz (2020). Available From: https://www.swissmedic.ch/swissmedic/de/home/news/coronavirus-covid-19/covid-19-impfstoff_erstzulassung.html (Accessed September 17, 2023).

[14] Federal Office of Public Health, Eidgenössische Kommission für Impffragen. Covid-19-Impfstrategie (2022). Bern Available From: www.bag.admin.ch/impfen-covid19 (Accessed September 17, 2023).

[15] Bergmann M Hannemann TV Bethmann A Schumacher AT. Determinants of SARS-CoV-2 Vaccinations in the 50+ Population (2021). Available From: https://www.ssrn.com/abstract=3938975 (Accessed September 17, 2023).

[16] Ruckstuhl L Czock A Haile SR Lang P. Influence of Cantonal Health Policy Frameworks & Activities on the Influenza Vaccination Rate in Patients With Non-Communicable Diseases in Switzerland. Vaccine (2022) 40(44):6326–36. 10.1016/j.vaccine.2022.09.038 36154757

[17] Petek D Kamnik-Jug K. Motivators and Barriers to Vaccination of Health Professionals Against Seasonal Influenza in Primary Healthcare. BMC Health Serv Res (2018) 18:853. 10.1186/s12913-018-3659-8 30428886 PMC6234642

[18] Arda B Durusoy R Yamazhan T Sipahi OR Taşbakan M Pullukçu H Did the Pandemic Have an Impact on Influenza Vaccination Attitude? A Survey Among Health Care Workers. BMC Infect Dis (2011) 11(1):87. 10.1186/1471-2334-11-87 21473763 PMC3084177

[19] Leonardelli M Mele F Marrone M Germinario CA Tafuri S Moscara L The Effects of the COVID-19 Pandemic on Vaccination Hesitancy: A Viewpoint. Vaccines (2023) 11(7):1191. 10.3390/vaccines11071191 37515007 PMC10386622

[20] MEDINSIDE. Mehrheit der Gesundheitsprofis Will Keine Rasche Corona-Impfung (2020). Available From: https://www.medinside.ch/de/post/corona-impfung-mehrheit-des-gesundheitspersonals-in-der-schweiz-ist-zurueckhaltend (Accessed September 17, 2023).

[21] Zürcher K Mugglin C Egger M Müller S Fluri M Bolick L Vaccination Willingness for COVID-19 Among Healthcare Workers: A Cross-Sectional Survey in a Swiss canton. Swiss Med Wkly (2021) 151(3738):w30061. 10.4414/SMW.2021.w30061 34546016

[22] Lang P Wu CTS Le-Nguyen AF Czock A. Influenza Vaccination Behaviour of Healthcare Workers in Switzerland: A Cross-Sectional Study. Int J Public Health (2023) 68:1605175. 10.3389/ijph.2023.1605175 36968266 PMC10036349

[23] Swiss Federal Statistical Office. Swiss Population by Language 2020 (2020). Available From: https://www.bfs.admin.ch/bfs/de/home/statistiken/bevoelkerung/sprachen-religionen.assetdetail.21344054.html (Accessed September 17, 2023).

[24] Swiss Federal Statistical Office. Swiss Population 2021 (2021). Available From: https://www.bfs.admin.ch/bfs/de/home/statistiken/bevoelkerung/erhebungen/bevnat.html (Accessed September 17, 2023).

[25] Federal Office of Public Health. Bericht zur Grippesaison 2021/22 (2022). Bern Available From: https://www.aramis.admin.ch/Dokument?DocumentID=69677 (Accessed September 17, 2023).

[26] Sani T Morelli I Sarti D Tassinari G Capalbo M Espinosa E Attitudes of Healthcare Workers Toward Influenza Vaccination in the COVID-19 Era. Vaccines (2022) 10(6):883. 10.3390/vaccines10060883 35746492 PMC9231023

[27] Zhelyazkova A Kim S Klein M Prueckner S Horster S Kressirer P COVID-19 Vaccination Intent, Barriers and Facilitators in Healthcare Workers: Insights From a Cross-Sectional Study on 2500 Employees at LMU University Hospital in Munich, Germany. Vaccines (2022) 10(8):1231. 10.3390/vaccines10081231 36016119 PMC9412572

[28] Kearns EC Callanan I O’Reilly A Purcell A Tuohy N Bulfin S Changing Attitudes Towards Annual Influenza Vaccination Amongst Staff in a Tertiary Care Irish University Hospital. Irish J Med Sci (2022) 191(2):629–36. 10.1007/s11845-021-02636-w 33987799 PMC8118105

[29] Krause C Steiger E Kroll LE Graf von Stillfried D Czihal TH Blitzumfrage COVID-19 Impfungen Online-Befragung (vom 05.08. bis 16.08.2021) Liefert Einblicke aus Vertragsarztpraxen in Deutschland zur Corona-Impfkampagne (2021). Available From: https://www.kv-thueringen.de/fileadmin/media2/Kommunikation/kvticker/Anhaenge/Zi-Paper_18-2021_Blitzumfrage_COVID-19_Impfungen.pdf (Accessed September 17, 2023).

[30] Trezzini B Meyer B Grez T Jans C Golder L Hohe Impfbereitschaft bei Ärztinnen und Ärzten. Schweizerische Ärztezeitung (2021) 102(44):1432–5. Available From: https://www.fmh.ch/files/pdf26/hohe-impfbereitschaft-bei-aerztinnen-und-aerzten.pdf (Accessed September 15, 2023).

[31] Willis GA Bloomfield L Berry M Bulsara C Bulsara M Chaney G The Impact of a Vaccine Mandate and the COVID-19 Pandemic on Influenza Vaccination Uptake in Western Australian Health Care Students. Vaccine (2022) 40(39):5651–6. 10.1016/j.vaccine.2022.08.040 36030122 PMC9393169

[32] Parker AM Atshan S Walsh MM Gidengil CA Vardavas R. Association of COVID-19 Vaccination With Influenza Vaccine History and Changes in Influenza Vaccination. JAMA Netw Open (2022) 5(11):e2241888. 10.1001/jamanetworkopen.2022.41888 36374504 PMC9664264

[33] Li K Yu T Seabury SA Dor A. Trends and Disparities in the Utilization of Influenza Vaccines Among Commercially Insured US Adults During the COVID-19 Pandemic. Vaccine (2022) 40(19):2696–704. 10.1016/j.vaccine.2022.03.058 35370018 PMC8960160

[34] Wang K Wong ELY Ho KF Cheung AWL Chan EYY Yeoh EK Intention of Nurses to Accept Coronavirus Disease 2019 Vaccination and Change of Intention to Accept Seasonal Influenza Vaccination During the Coronavirus Disease 2019 Pandemic: A Cross-Sectional Survey. Vaccine (2020) 38(45):7049–56. 10.1016/j.vaccine.2020.09.021 32980199 PMC7834255

[35] Federal Office of Public Health. COVID-19 Vaccination Data From Swiss Federal Health Office (2023). Available From: https://www.covid19.admin.ch/en/overview. (Accessed September 17, 2023).

[36] Krause C Steiger E Kroll LE von Stillfried DG Czihal T Blitzumfrage COVID-19 impfungen online-befragung (vom 05.08. bis 16.08.2021) liefert Einblicke aus Vertragsarztpraxen in Deutschland zur Corona-Impfkampagne. Zi-Paper (2021).

[37] Askarian M Semenov A Llopis F Rubulotta F Dragovac G Pshenichnaya N The Covid-19 Vaccination Acceptance/Hesitancy Rate and Its Determinants Among Healthcare Workers of 91 Countries: A Multicenter Cross-Sectional Study. EXCLI J (2022) 21:93–103. 10.17179/excli2021-4439 35221837 PMC8859647

[38] Gagneux-Brunon A Detoc M Bruel S Tardy B Rozaire O Frappe P Intention to Get Vaccinations Against COVID-19 in French Healthcare Workers During the First Pandemic Wave: A Cross-Sectional Survey. J Hosp Infect (2021) 108:168–73. 10.1016/j.jhin.2020.11.020 33259883 PMC7699157

[39] Heiniger S Schliek M Moser A Von Wyl V Höglinger M. Differences in COVID-19 Vaccination Uptake in the First 12 Months of Vaccine Availability in Switzerland – A Prospective Cohort Study. Swiss Med Wkly (2022) 152(1314):w30162. 10.4414/SMW.2022.w30162 35429238

[40] Federal Office of Public Health. BAG Bulletin 28/15 (2015). Bern Available From: https://www.bag.admin.ch/bag/de/home/das-bag/publikationen/periodika/bag-bulletin.html (Accessed September 17, 2023).

[41] Karlsson LC Lewandowsky S Antfolk J Salo P Lindfelt M Oksanen T The Association Between Vaccination Confidence, Vaccination Behavior, and Willingness to Recommend Vaccines Among Finnish Healthcare Workers. PLoS One (2019) 14(10):e0224330. 10.1371/journal.pone.0224330 31671115 PMC6822763

[42] Jun J Tubbs Cooley H O’Mathúna DP Kim M Pignatiello G Fitzpatrick JJ Individual and Work-Related Characteristics Associated With COVID-19 Vaccination Status Among Ohio Nurses. Policy Polit Nurs Pract (2023) 24(2):81–90. 10.1177/15271544221141060 36482714 PMC9742733

[43] Jiang SQ Cai YW Zuo R Xu LF Zheng JD Yi HY Analysis of Influenza Vaccination Coverage, Recommendation Behaviors and Related Factors Among Health Care Workers in Nanshan District of Shenzhen City Under the Free Policy Between 2019 and 2020. Zhonghua Yu Fang Yi Xue Za Zhi (2022) 56(11):1565–70. 10.3760/cma.j.cn112150-20211217-01164 36372745

[44] Zürcher K Zwahlen M Berlin C Egger M Fenner L. Losing Ground at the Wrong Time: Trends in Self-Reported Influenza Vaccination Uptake in Switzerland, Swiss Health Survey 2007–2017. BMJ Open (2021) 11(2):e041354. 10.1136/bmjopen-2020-041354 PMC7875279 33563620

[45] Rieck T Steffen A Schmid-Küpke N Feig M Wichman O Siedler A. Impfquoten bei Erwachsenen in Deutschland-Aktuelles aus der KV-Impfsurveillance und der Onlinebefragung von Krankenhauspersonal OKaPII 1. Epidemiologisches Bull (2020) 47:3–26. 10.25646/7658

[46] Kassianos G Kuchar E Nitsch-Osuch A Kyncl J Galev A Humolli I Motors of Influenza Vaccination Uptake and Vaccination Advocacy in Healthcare Workers: A Comparative Study in Six European Countries. Vaccine (2018) 36(44):6546–52. 10.1016/j.vaccine.2018.02.031 29605515

[47] Meckawy R Ghare F Cabrera M Thabius A Moore M Lomazzi M. Impact of COVID-19 Pandemic on Health Workers Sentiment Towards Influenza Vaccination: A Literature Review. Popul Med (2023) 5:1–13. 10.18332/popmed/162880

[48] Papageorgiou C Mazeri S Karaiskakis M Constantinou D Nikolaides C Katsouris S Exploring Vaccination Coverage and Attitudes of Health Care Workers Towards Influenza Vaccine in Cyprus. Vaccine (2022) 40(12):1775–82. 10.1016/j.vaccine.2022.02.020 35168841

[49] Halpin C Reid B. Attitudes and Beliefs of Healthcare Workers About Influenza Vaccination. Nurs Old People (2019) 31(2):32–9. 10.7748/nop.2019.e1154 31468782

[50] Briko NI Korshunov VA Mindlina AY Polibin RV Antipov MO Brazhnikov AI Healthcare Workers’ Acceptance of COVID-19 Vaccination in Russia. Int J Environ Res Public Health (2022) 19(7):4136. 10.3390/ijerph19074136 35409818 PMC8998926

[51] Pavlič DR Maksuti A Podnar B Kokalj Kokot M. Reasons for the Low Influenza Vaccination Rate Among Nurses in Slovenia. Prim Health Care Res Dev (2020) 21:e38. 10.1017/S1463423620000419 32993841 PMC7576542

[52] Karafillakis E Dinca I Apfel F Cecconi S Wűrz A Takacs J Vaccine Hesitancy Among Healthcare Workers in Europe: A Qualitative Study. Vaccine (2016) 34(41):5013–20. 10.1016/j.vaccine.2016.08.029 27576074

[53] Lorenc T Marshall D Wright K Sutcliffe K Sowden A. Seasonal Influenza Vaccination of Healthcare Workers: Systematic Review of Qualitative Evidence. BMC Health Serv Res (2017) 17(1):732. 10.1186/s12913-017-2703-4 29141619 PMC5688738

[54] Millner VS Eichold BH Franks RD Johnson GD. Influenza Vaccination Acceptance and Refusal Rates Among Health Care Personnel. South Med J (2010) 103(10):993–8. 10.1097/SMJ.0b013e3181eda3d5 20818310

[55] Kose S Mandiracioglu A Sahin S Kaynar T Karbus O Ozbel Y. Vaccine Hesitancy of the COVID‐19 by Health Care Personnel. Int J Clin Pract (2021) 75(5):e13917. 10.1111/ijcp.13917

[56] Rapisarda V Vella F Ledda C Barattucci M Ramaci T. What Prompts Doctors to Recommend COVID-19 Vaccines: Is It a Question of Positive Emotion? Vaccines (2021) 9(6):578. 10.3390/vaccines9060578 34205935 PMC8229710

[57] Ammeter T Lang P Czock A. Overview of the Influenza Vaccination Activities and Legal Frameworks in 26 Swiss Cantons During the Influenza Season 2019/20. Vaccine (2022) 40(12):1702–6. 10.1016/j.vaccine.2022.02.009 35172940

[58] pharmasSuisse. Impfen und Impfberatung (2023). Available From: https://www.pharmasuisse.org/de/1159/Impfen-und-Impfberatung.htm (Accessed September 17, 2023).

[59] Federal Office of Public Health. Apothekerinnen und Apotheker 2022 (2023). Available From: file:///C:/Users/20121/Downloads/Apothekerinnen_und_Apotheker_2022_d.pdf (Accessed September 15, 2023).

[60] Hostettler S Kraft E. FMH-Ärztestatistik 2020 – die Schweiz im Ländervergleich (2020). Bern Available From: https://www.fmh.ch/files/pdf28/saez-artikel-fmh-aerztestatistik-2020-de.pdf (Accessed September 17, 2023).

[61] Merçay C Grünig A Dolder P. Gesundheitspersonal in der Schweiz – Nationaler Versorgungsbericht 2021 Bestand, Bedarf, Angebot und Massnahmen zur Personalsicherung (2021). Bern. Available From: https://www.gdk-cds.ch/fileadmin/docs/public/gdk/themen/gesundheitsberufe/nichtun._gesundheitsberufe/versorgungsbericht/Obsan_03_2021_BERICHT-D_korr_def.pdf (Accessed September 17, 2023).

[62] Papis T Clavien C. Do Primary Care Physicians Contribute to the Immunization Status of Their Adult Patients? A Story of Patients’ Overconfidence Coupled With Physicians’ Passivity. Front Med (2021) 8:655734. 10.3389/fmed.2021.655734 PMC8245703 34222277

[63] Federal Office of Public Health. What Is the Covid Certificate? (2023). Available from: https://foph-coronavirus.ch/certificate/what-is-the-covid-certificate (Accessed September 17, 2023).

