# Supplementary material for: Peri-Pandemic Acceptance of Influenza and COVID-19 Vaccination by Swiss Healthcare Workers in Primary Care 2020/21: A Cross-Sectional Study
Source: Int J Public Health. 2023 Nov 15;68:1605832. doi: 10.3389/ijph.2023.1605832 (PMC10684700; doi:10.3389/ijph.2023.1605832)
Supplement: Supplementary file 1 [file DataSheet2.pdf]

Supplementary Figure 1

Part. A Reasons (%) of HCWs for getting vaccinated against the influenza, detailed by language

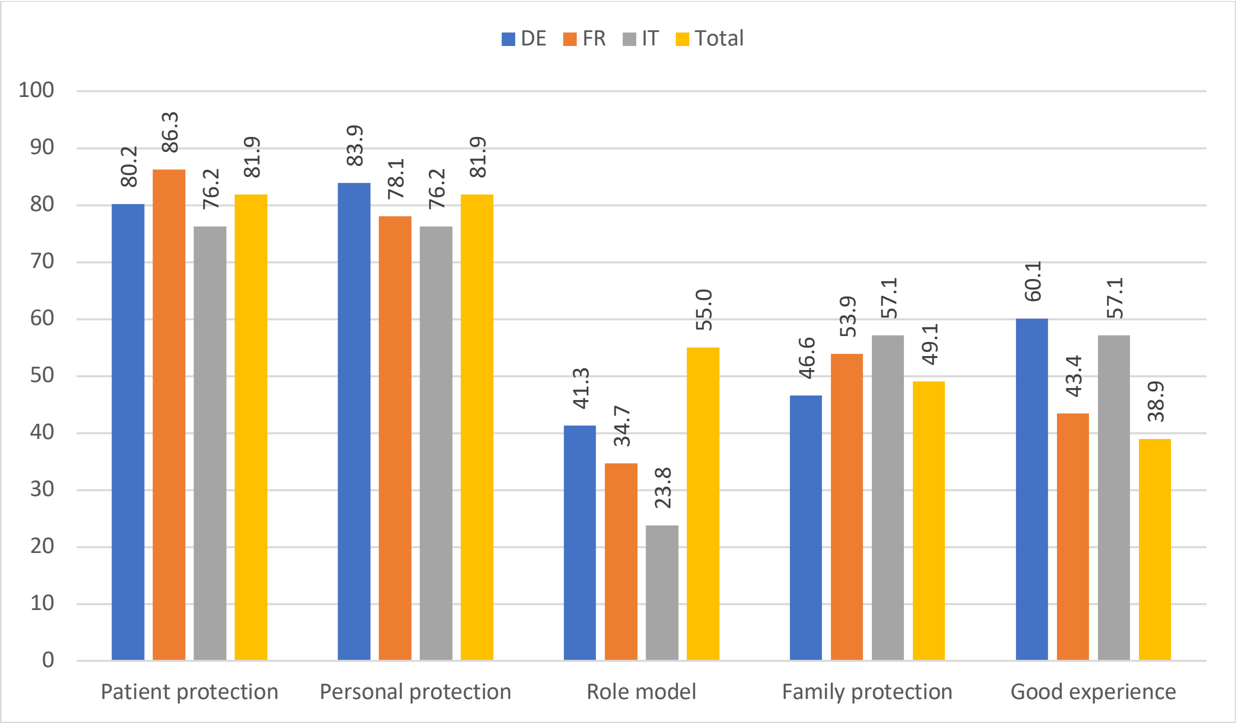

Multiple responses were possible. HCWs- healthcare workers. DE -German; FR-French; IT-Italian.

Part B. Reasons (%) of HCWs for getting vaccinated against COVID-19, detailed by language

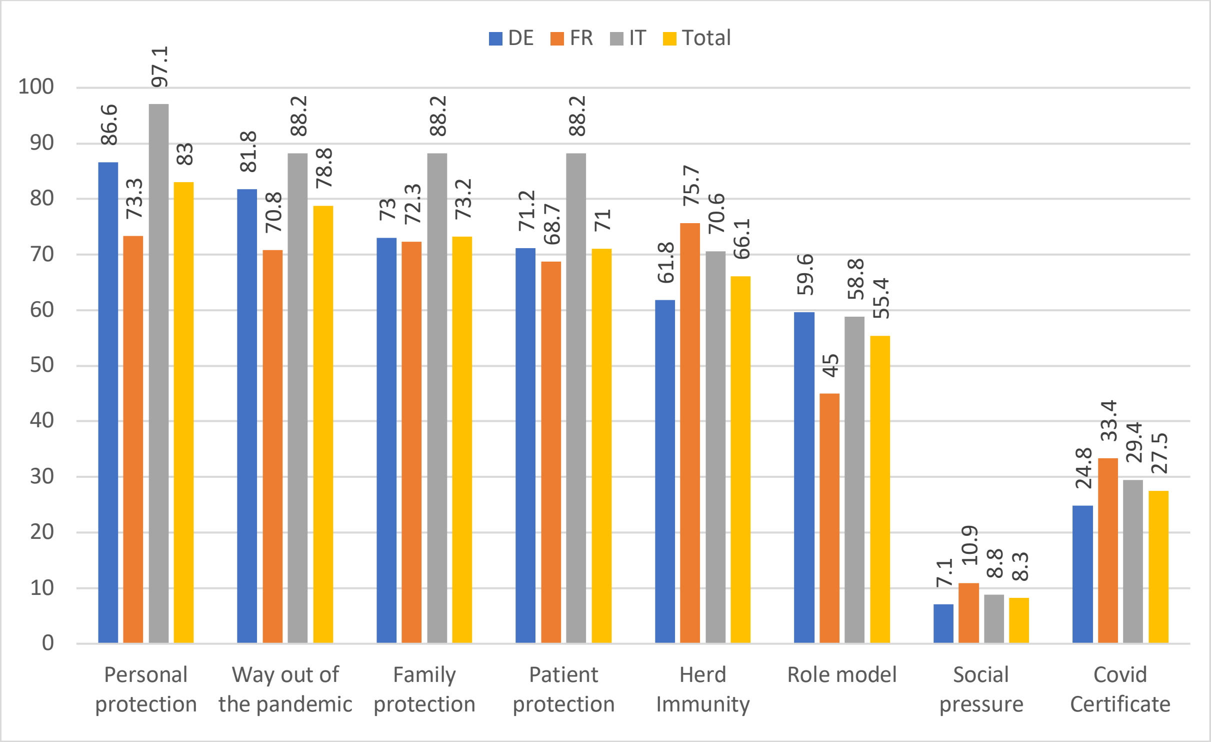

Multiple responses were possible. HCWs- healthcare workers. DE -German; FR-French; IT-Italian.

Supplementary Figure 2

Part A. Reasons (%) of HCWs for recommending the influenza vaccination to patients/clients, detailed by language

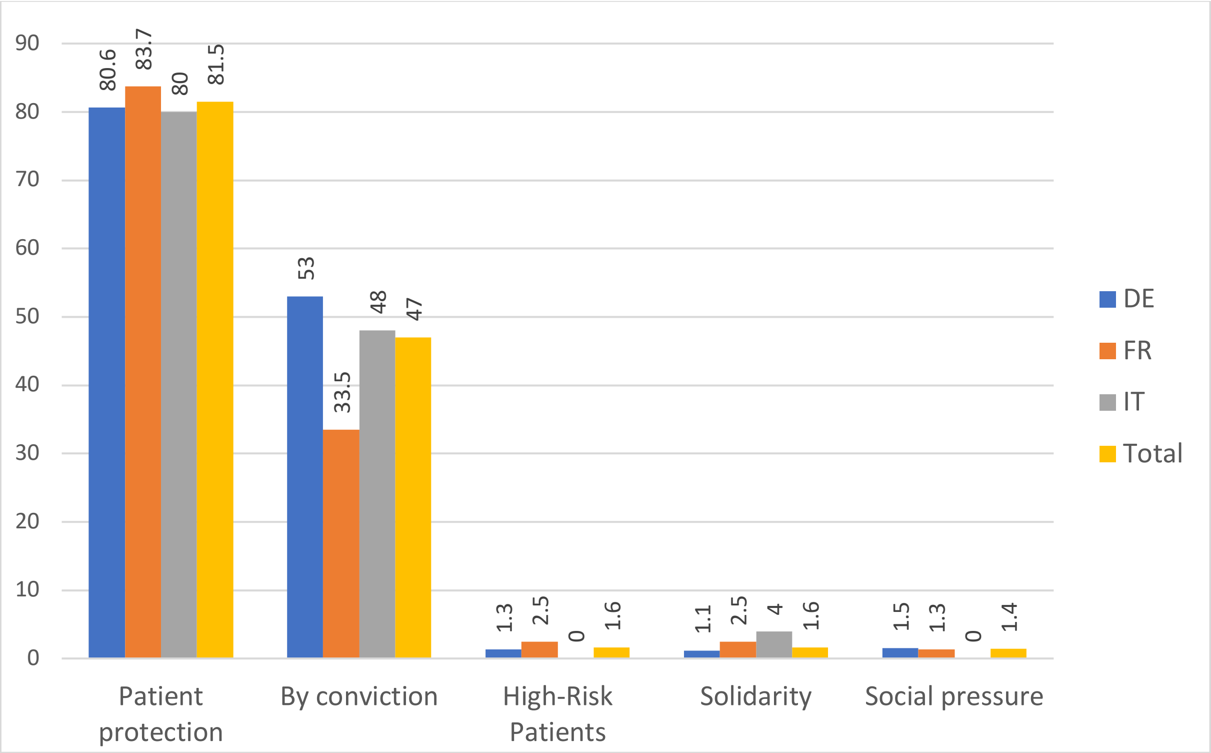

Multiple responses were possible. HCWs- healthcare workers. DE -German; FR-French; IT-Italian.

Part B Reasons (%) of HCWs for recommending the COVID-19 vaccination to patients/ clients, detailed by language

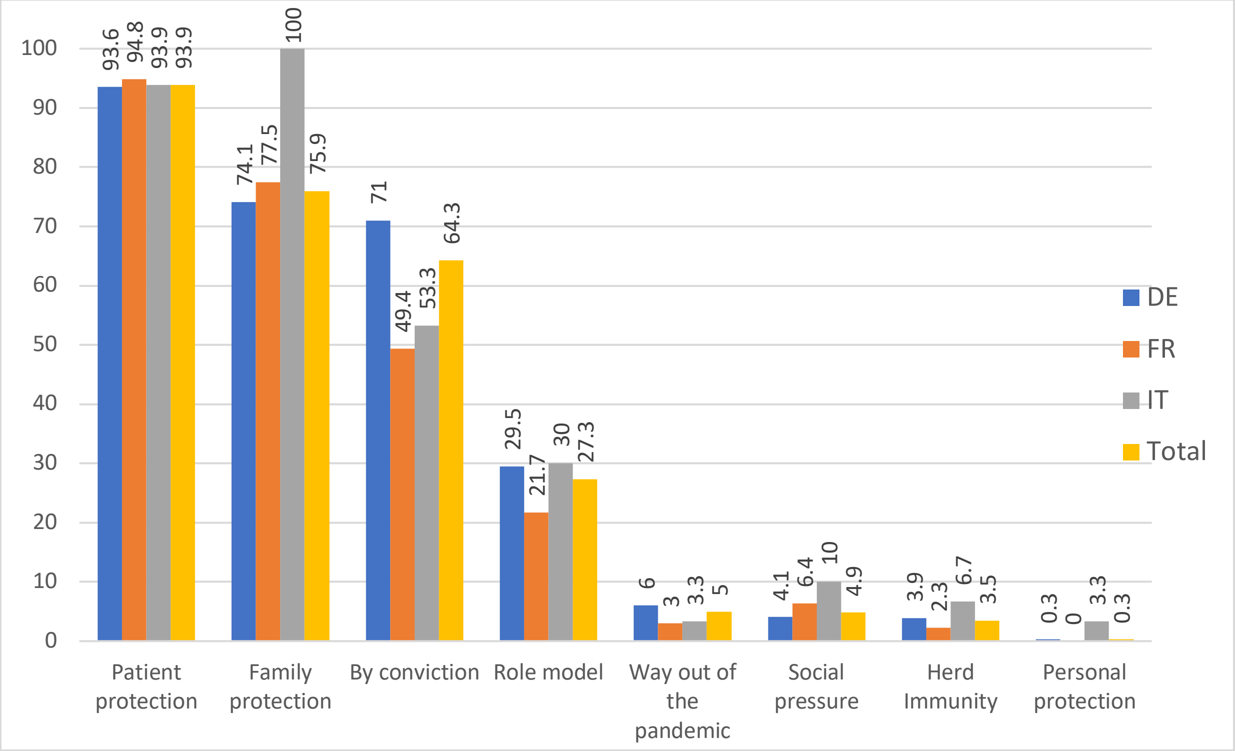

Multiple responses were possible. HCWs- healthcare workers. DE -German; FR-French; IT-Italian.

Supplementary Table 1

Reasons (%) of HCWs for not getting vaccinated against influenza and COVID-19

**Part A. Reasons against influenza vaccination**	**Frequency**	**% of Cases**
I am not in the vulnerable group	212	45.40
I trust my immune system	193	41.33
Personal isolation in case of infection	105	22.48
Hygiene measures/ mouth protection are sufficient	64	13.70
No flu epidemic	58	12.42
Flu is usually harmless	51	10.92
Vaccination is not effective	40	8.57
Got the flu due to vaccination	38	8.14
Common side effects	38	8.14
No flu vaccination yet	29	6.21
Forgot	26	5.57
Increase susceptibility to infections	25	5.35
Self-determination	20	4.28
Flu despite vaccination	15	3.21
Distrust of pharmaceutical industry	9	1.93
Allergy	6	1.28
Patient should vaccinate themselves	5	1.07
Too little research on the flu vaccination	2	0.43
Risk of infection from a third person	1	0.21
**Total**	**954**	

Multiple responses were possible. HCWs- healthcare workers.

Supplementary Table 1

**Part B. Reasons against COVID-19 vaccination**	**Frequency**	**% of Cases**
Vaccination is insufficiently researched	64	65.31
Vaccination is not effective	54	55.10
I am not in the vulnerable group	53	54.08
I trust my immunity	52	53.06
Long-term damage	49	50.00
Distrust the new mRNA technology	47	47.96
Common side effects	43	43.88
Previous COVID-19 Infection	32	32.65
Distrust of pharmaceutical industry	30	30.61
Rare severe side effects	29	29.59
Self-determination	24	24.49
Hygiene measures are sufficient	20	20.41
Medical reasons (e.g., allergies)	16	16.33
Fear of infertility/ sterility	11	11.22
Self-isolation in case of infection	7	7.14
Pregnancy	4	4.08
**Total**	**535**	

Multiple responses were possible.

Supplementary Table 2 The influence (%) of the COVID-19 pandemic on the decision of HCWs to get vaccinated against the influenza

Did the pandemic influence their decision to vaccinate?	Total	Percent	Sex, %			Language, %		
	n	%	F	M	O	DE	FR	IT
No	874	70.7	70.2	73.7	50.0	71.6	67.9	77.1
Yes, little	210	17.0	16.9	17.4	25.0	17.5	16.3	11.4
Yes, very much	129	10.4	10.6	9.0	25.0	9.5	12.5	11.4
Do not know	24	1.9	2.3	0.00	0.00	1.4	3.3	0.00
**Total**	**1237**		**Pearson chi2(6) = 6.4205 Pr = 0.378**			**Pearson chi2(6) = 8.9123 Pr = 0.179**		

F-female, M-male, O-others; DE- German, FR-French, IT-Italian. HCWs- healthcare workers; n-number of participants. Total n = 1237. % - Percent.

Supplementary Table 3. Reasons (%) of HCWs against recommending influenza and COVID-19 vaccination to their patients/clients

Part A. Influenza	n	% of Cases	Part B. COVID-19	n	% of Cases
Matter of the doctor	18	52.9	*Private matter*	29	64.4
Private matter	14	41.2	*I am not convinced*	19	42.2
I am not convinced	10	29.4	*Matter of the doctor*	15	33.3
I do not vaccinate myself	3	8.8	*I do nat vaccinate myself*	7	15.6
No patients	0	0.00	*No patients*	0	0.00
**Total**	**45**		**Total**	**70**	
