# Supplementary material for: Peri-Pandemic Acceptance of Influenza and COVID-19 Vaccination by Swiss Healthcare Workers in Primary Care 2020/21: A Cross-Sectional Study
Source: Int J Public Health. 2023 Nov 15;68:1605832. doi: 10.3389/ijph.2023.1605832 (PMC10684700; doi:10.3389/ijph.2023.1605832)
Supplement: Supplementary file 2 [file DataSheet1.pdf]

STANDARDISED ONLINE QUESTIONNAIRE ON VACCINATION ACCEPTANCE AND RATE OF VACCINATION AGAINST SEASONAL INFLUENCE AND SARS-COV-2 IN HEALTH CARE PERSONS IN PRIMARY CARE.

Influenza vaccination is a controversial topic among many. In view of the current COVID 19 pandemic and its further development, we are interested in the vaccination rate and vaccination acceptance among health care workers, especially in contact with patients with chronic diseases. What reasons are given for not getting vaccinated? We are also interested in the same questions in connection with the COVID-19 vaccination. By participating in the survey, you agree that your answers will be evaluated **anonymously** and that the results of the survey will be published in aggregated form. The answers are **anonymous** and can therefore neither be deleted nor filtered out or tracked.

Thank you for your participation! You will help us to better evaluate vaccination preparedness for both diseases and the current communication about vaccination.

Demographics

Demographics

Age: Birthyear ___________________

Gender: ☐ male ☐ female ☐ other

Function/profession:

☐ physician
☐ pharmacist
☐ other medical professional according to the Swiss Law of Medical Professions (dentist, chiropractor, veterinarian)
☐ registered nurse
☐ other health professional / therapist (Physiotherapist, nutritionist, diabetes nurse, ..)
☐ medical practice assistant
☐ pharmacy technician
☐ psychosocial therapist
☐ Administration
☐ Prevention / Public Health
☐ other ……

Place of work

☐ Hospital
☐ Practice (general practitioner, dentist, chiropractor, physiotherapy, psychology, etc.)
☐ Spitex

- ☐ Homecare (Heimeim)
- ☐ Rehabilitation
- ☐ Pharmacy
- ☐ Counselling centre
- ☐ Prevention / Public Health
- ☐ Pharmaceutical industry
- ☐ Management / Administration
- ☐ Others_______________

Working canton, incl. FL

- ☐ Aargau
- ☐ Appenzell AI
- ☐ Appenzell AR
- ☐ Basel-Landschaft
- ☐ Basel-Stadt
- ☐ Bern
- ☐ Freiburg
- ☐ Geneva
- ☐ Glarus
- ☐ Grisons
- ☐ Jura
- ☐ Lucerne
- ☐ Neuchâtel
- ☐ Nidwalden
- ☐ Obwalden
- ☐ St. Gallen
- ☐ Schaffhausen
- ☐ Schwyz
- ☐ Solothurn
- ☐ Ticino
- ☐ Thurgau
- ☐ Uri
- ☐ Zurich
- ☐ Zug
- ☐ Valais
- ☐ Vaud
- ☐ Liechtenstein

Questions about the flu vaccination

1. Did you get vaccinated against seasonal flu last autumn/winter (2020/21)?

☐ Yes ☐ No

If yes: Why?

- ☐ Patient protection
- ☐ Self-protection
- ☐ Good experience with prior vaccination
- ☐ For the protection of family members
- ☐ Role model
- ☐ Other reasons, namely :...

Multiple answers possible

If no: Why not?

- ☐ Common side effects
- ☐ Possibility of vaccine damage
- ☐ Increases my susceptibility to infections
- ☐ Flu-like illness due to vaccination
- ☐ Flu illness despite vaccination
- ☐ Hygiene measures and mouth protection are sufficient
- ☐ There was no flu epidemic at all last winter (2020/21)
- ☐ Vaccination does not work / works too little
- ☐ I do not want to be told what to do / self-determination
- ☐ The relatives / visitors are the problem (contagious)
- ☐ Flu is usually harmless
- ☐ I do not belong to the vulnerable group
- ☐ I stay at home when I am ill
- ☐ I trust my immune system / Flu is a good workout for the immune system
- ☐ Patients should vaccinate themselves
- ☐ Too little research on the Flu vaccination
- ☐ I have great distrust of the pharmaceutical industry
- ☐ Other reasons, namely :...

Multiple answers possible

2. Do you recommend seasonal flu vaccination to your patients/clients?

☐ Yes ☐ No ☐ Only in certain cases

If yes: Why?

- ☐ out of conviction
- ☐ to protect my patients/clients
- ☐ Other reasons, namely: ....

If no: Why not?

- ☐ I am not convinced about the flu vaccination
- ☐ I do not get vaccinate myself
- ☐ This is a private matter / self-determination
- ☐ This is a matter for the treating doctor
- ☐ Other reasons, namely: ....

If only in certain cases: In which cases?

- ☐ For patients at risk
- ☐ When I get asked about it
- ☐ Other reasons, namely :...

3. **Has the spread of COVID - 19 infection influenced your decision to have prophylactic flu vaccination in autumn/winter 2020/21?**

☐ Yes, very much ☐ Yes, a little bit ☐ No ☐ Don't know

4. **Will you get vaccinated against influenza in autumn/winter 2021/22?**

☐ Yes ☐ No ☐ Do not know

Questions about Covid-19 vaccination

5. Have you been vaccinated against Covid - 19?

☐ Yes ☐ No

If yes:

a. When? Month / Year______________________

b. With how many doses?

☐ one ☐ two ☐ three

If only one dose, why:

☐ Have had the disease
☐ Had too many side effects
☐ Was critical of the vaccine from the start
☐ Medical reasons

c. **Why did you get vaccinated?**

☐ Herd immunity
☐ Patient protection
☐ Self-protection
☐ For the protection of family members
☐ Vaccination as the fastest way out of the pandemic
☐ Role model function
☐ Social pressure
☐ To get a Covid certificate
☐ Other reasons, namely:________________

Multiple answers possible

d. With which vaccine?

☐ mRNA
☐ other

e. **Have you had any side effects yourself?**

☐ Yes, more than expected
☐ Yes, as expected
☐ Yes, but less bad than expected
☐ No

____

If you are NOT vaccinated: Why not?

☐ Previous Covid 19 infection
☐ I do not belong to the vulnerable group
☐ I trust my immune system
☐ Vaccination does not work: Covid-19 infection possible despite vaccination
☐ due to frequent side effects
☐ due to rare severe side effects
☐ because of possible long-term damage
☐ I don't trust the new mRNA technology
☐ Vaccination has been too little researched
☐ I have great distrust of the pharmaceutical industry
☐ Hygiene measures and mask protection are sufficient
☐ Self-isolation / quarantine is sufficient
☐ I don't let anyone tell me what to do /self-determination
☐ Pregnancy
☐ Fear of infertility
☐ Medical reasons (e.g. allergies)
☐ Other reasons, namely: ....

Multiple answers possible

6. Do you already have Covid-19?

☐ Yes ☐ No ☐ Do not know

If yes, when? Month/Year________________________

7. Do you recommend COVID-19 vaccination to your patients/clients?

☐ Yes ☐ No ☐ Only in certain cases

If yes: Why?

☐ Patient protection
☐ Self-protection
☐ Out of conviction
☐ For the protection of patients and family members
☐ Role model function
☐ Social pressure
☐ Other reasons, namely:
....

Multiple answers possible

If no: Why not?

- ☐ Because I am not convinced of the COVID-19 vaccination
- ☐ Because I do not vaccinate myself
- ☐ This is a private matter / self-determination
- ☐ This is a matter for the doctor treating you
- ☐ Other reasons, namely: ....

If only in certain cases: In which cases?

- ☐ For at-risk patients/clients
- ☐ When I am asked about it
- ☐ Other reasons, namely: ....

8. Are you trained to vaccinate?

- ☐ Yes
- ☐ I am currently being trained
- ☐ No, but I am considering / planning to do vaccination training
- ☐ No
